# Extracellular vesicles in neuroblastoma: role in progression, resistance to therapy and diagnostics

**DOI:** 10.3389/fimmu.2024.1385875

**Published:** 2024-04-09

**Authors:** Mayura R. Dhamdhere, Vladimir S. Spiegelman

**Affiliations:** Division of Pediatric Hematology and Oncology, Department of Pediatrics, The Pennsylvania State University College of Medicine, Hershey, PA, United States

**Keywords:** neuroblastoma, extracellular vesicles, NB TME, pre-metastatic niche, therapeutic resistance, diagnosis and prognosis

## Abstract

Neuroblastoma (NB) is the most common extracranial solid pediatric cancer, and is one of the leading causes of cancer-related deaths in children. Despite the current multi-modal treatment regimens, majority of patients with advanced-stage NBs develop therapeutic resistance and relapse, leading to poor disease outcomes. There is a large body of knowledge on pathophysiological role of small extracellular vesicles (EVs) in progression and metastasis of multiple cancer types, however, the importance of EVs in NB was until recently not well understood. Studies emerging in the last few years have demonstrated the involvement of EVs in various aspects of NB pathogenesis. In this review we summarize these recent findings and advances on the role EVs play in NB progression, such as tumor growth, metastasis and therapeutic resistance, that could be helpful for future investigations in NB EV research. We also discuss different strategies for therapeutic targeting of NB-EVs as well as utilization of NB-EVs as potential biomarkers.

## 1 Introduction - NB and EVs

### 1.1 Neuroblastoma

Neuroblastoma (NB) is a common extracranial solid tumor in children that accounts for around 15% of cancer-related mortalities in pediatric patients (1–4). NB originates from the progenitor cells of the sympathetic nervous system, leading to tumors in the adrenal glands and/or sympathetic ganglia along the paravertebral chain (4). Moreover, NB is characterized by a broad spectrum of clinical variability ranging from spontaneous regression of some tumors to therapeutic resistance and relapse of others (2). The International Neuroblastoma Risk Group (INRG) system thus classifies tumors based on a combination of clinical and biological markers such as patient age, histologic category, stage, grade of differentiation, *MYCN* amplification, chromosome 11q status and DNA ploidy to guide risk-based treatments for patients with NB (3). Low-risk group patients generally have an excellent disease outcome with >95% overall survival with surgical resection. Also, a subset of younger patients within the low-risk group even demonstrate spontaneous regression of tumors without any treatment (1, 3). Likewise, patients with intermediate-risk NBs have also shown favorable prognosis with surgery and/or chemotherapy (1, 3). However, the high-risk (HR) group, characterized by distant metastasis and age older than 18 months, have long-term survival of only 40-50% (3). Despite recent multimodal treatment regimens which include induction chemotherapy, consolidation therapy (including high dose chemo + autologous stem-cell transplant and irradiation) and immunotherapy, 5-year survival in the HR group still remains low (<50%) (1, 2). Importantly, around half of all NB patients are classified to have HR disease with evident distant site metastasis (2, 3, 5, 6), and majority of which develop recurrence (5, 7). Metastasis and therapeutic resistance are thus major challenges in HR-NB treatment, leading to poor disease outcomes (7, 8), thus indicating the need for identifying unconventional and novel therapeutic targets for the treatment of metastatic, recurrent NBs.

Besides the commonly associated MYCN amplification (~20-25%) (2) and 11q loss (~35-45%) (9, 10), other well-known oncogenic drivers of NB include chromosomal abnormalities such as 1p loss and 17q gain that have been individually associated with poor outcomes (11). Importantly, 17q gain is found in more than 50% of all NB cases, and is independently associated with poor survival in patients with advanced-stage-NB (11–13), thus suggesting its clinical significance in NB. Several candidate oncogenes found on this frequently amplified 17q21-ter locus (14) are being investigated for their relevance in NB progression. One of these candidate oncogenes, IGF2BP1, was shown to promote NB metastasis by altering the function of NB-secreted small extra cellular vesicles (EVs) (15). Besides, the importance of EVs in NB pathogenesis and resistance to therapies have also been recently demonstrated. Given the aggressive nature of NB, and the importance of EVs in cancer, in this review, we summarize current knowledge of the role EVs play in NB progression and opportunities it presents for therapeutic modalities.

### 1.2 Metastasis and extracellular vesicles

Metastasis – the primary cause of death in majority of cancers including NB, is a multistage process that is regulated by the microenvironment at the primary tumor location as well as secondary sites. The tumor microenvironment (TME) at the primary site consists of a highly dynamic extracellular matrix (ECM), stromal and immune cells, several secreted factors such as cytokines, chemokines and extracellular vesicles, which all collectively modulate the motility, dissemination, and colonization of the cancer cells at secondary locations (16). Primary tumors induce changes at the potential secondary organs by creating a favorable environment for cancer cells to colonize and initiate metastasis – a phenomenon referred to as “pre-metastatic niche” (PMN) (17, 18). PMN formation is marked by remodeling of the tissue microenvironment to a dysfunctional, tumor-promoting milieu which includes several step-wise events like vascular leakiness, extracellular matrix (ECM) remodeling and induction of an immunosuppressive environment at the secondary location (18). Several tumor secreted factors, in particular small extracellular vesicles (EVs) have been shown to induce the formation of PMN (19, 20) thereby priming these secondary sites for future metastasis.

Extracellular vesicles are a class of heterogeneously sized particles released by all actively proliferating cells for intercellular communication. These secreted vesicles comprise majorly of two types – i) plasma membrane-shed microvesicles (100 to 1000 nm), and ii) small extracellular vesicles (sEVs) (< 200 nm) either derived from the endosomal system (also known as exosomes) or from the plasma membrane (21–26). Initially identified as a disposal mechanism of cells (27), studies in the last few decades have demonstrated the significance of these vesicles as signal mediators in normal physiological as well as diseased conditions (22, 28). Their vesicular cargo consists of various biomolecules including proteins, nucleic acids (mRNA, DNA, long non-coding RNA and miRNAs), lipids, metabolites and glycans, that influence the phenotype of the recipient cells. Since a large number of studies have reported endosome-derived small extracellular vesicles (referred to as EVs here on) as major intercellular communicators in local and systemic environments, in various diseases and importantly in cancer progression, these specific endosomal-origin EVs are thus a major focus of our review.

EVs are 30-150 nm sized vesicles generated by the inward budding of the late endosomal membrane, forming a pool of intraluminal vesicles (ILV) within the multivesicular bodies (MVBs). Fusion of MVBs with the plasma membrane ultimately releases these EVs into the extracellular space. Biogenesis of EVs include a series of events and related proteins such as Rab GTPases that control endosomal trafficking; the endosomal sorting complex for transport (ESCRT) proteins which mediate ILV formation; transmembrane proteins like tetraspanins that induce membrane curvature; and sphingomyelinases that produce ceramides which are essential for vesicle formation (21, 23, 29). However, the exact mechanism of EV biogenesis is still an area of debate and a clear consensus on specific endosome origin markers is lacking. Hence, International society for extracellular vesicles (ISEV) recommends utilizing a combination of factors such as size and expression of biomarkers including tetraspanins (CD9, CD63 and CD81), ESCRT associated proteins - Alix and TSG101, Syntenin-1 and Flotillin-1 to characterize small extracellular vesicles (EVs) (24). Various isolation methods including ultracentrifugation, density gradient centrifugation and various precipitation techniques have been described to isolate EVs from cell culture supernatants and clinical samples (30). Several quantifying techniques have been used for EV analysis, like Nanoparticle tracking analysis (NTA), Dynamic light scattering (DLS), transmission electron microscopy (TEM), several immunodetection methods, Mass spectrometry based proteomic analysis and etc. (30). ISEV recommends using more than one type of these analysis methods for EV validation (24).

There have been a plethora of studies reporting the importance of EVs and their diverse functions at various stages of tumor progression in multiple cancer types, however much less was known about the role of EVs in NB. Several studies have emerged in the past few years indicating the involvement of EVs in various aspects of NB progression. This review encompasses such recent findings and advances involving EVs in NB tumor progression, metastasis, regulation of the tumor microenvironment (TME) and tissue microenvironment, pre-metastatic niche formation, imparting therapeutic resistance, and their potential application in NB diagnosis/prognosis, thus providing with an opportunity to envision the utilization of EVs for developing novel therapeutic strategies for treating NBs.

## 2 Tumor derived EVs

Accumulation of genetic abnormalities result in the formation of neoplastic cells, however, several factors in the surrounding or tissue microenvironment are essential for the manifestation of a tumor. The TME is a complex ecosystem instructed by the cancer cells, that is composed of cellular and acellular components, contributed by both tumor as well as host cells. TME is a dynamic network of interactions between its components like fibroblasts, vascular endothelial cells, immune cells, adipocytes, pericytes and various other tissue-resident cells, embedded in an altered ECM (16, 31). This inter-cellular communication is mediated by several secretory components like growth factors, cytokines, chemokines and extracellular vesicles. Different factors of the TME thus ensure the growth and progression of tumor by maintaining the homeostasis of the tumor cells, hampering the immune surveillance and efficient drug carting (31, 32).

In addition to local signaling within the primary TME, tumor cells regulate the distant future metastatic sites to form a metastasis favorable environment, by transporting bioactive molecules through EVs. Tumor cells secrete enormous amounts of EVs that have known to play diverse functions in promoting overall cancer progression in several cancers (20). Tumor derived EVs (TEVs) promote neoplasia & EMT, metastasis, regulate the immune system, induce PMN, impart oncogenic properties and therapeutic resistance by regulating other tumor cells and components of the microenvironment (ME) like stromal/immune cells. TEV-cargo consists of a variety of signaling molecules like DNA, mRNA, miRNAs, other small non-coding RNA species, and proteins that are transferred to the target cells in the ME, thereby altering the recipient cell phenotype (19, 20, 33). For example, TEVs from hypoxic colorectal cancer cells (CRC) were found to transfer oncogenic signals to the less aggressive normoxic cancer cells thereby increasing survival and proliferation ability of the recipient cells (34). Lu et al., 2021 showed the function of TEVs in breast cancer progression, by transporting miR-182-5p microRNA to endothelial cells, and enhancing their proliferation and migration, thereby promoting angiogenesis (35). Another study reports the effect of EV-circRNA (cicCOL1A1) in promoting angiogenesis of HUVECs by EIF4A3 mediated activation of Smad2/3 pathway, in CRC (36).

Similarly, numerous other studies have indicated the implication of TEVs in immune evasion. Of which, a few notable examples of studies demonstrate the role of TEVs in mediating immune suppression, by inducing the polarization of neutrophils towards a pro-tumor N2-like phenotype (37), by stimulating the differentiation of monocytes to tumor-supporting macrophages (38–40), and by promoting immunosuppressive lymphocyte signature (41) in multiple cancer models. Moreover, Xie et al., 2022 showed that breast cancer cell-derived EVs induced exhaustion of CD8^+^ T cells via TGF-β-SMAD3-TCF1 signaling (42). While CRC-derived EVs were found to induce M2 like polarization of the recipient macrophages, by upregulating the expression of PD-L1 via PTEN/AKT and SCOS1/STAT1 pathways in these cells, and ultimately affecting the T cell activity in the CRC TME (43). Similarly, melanoma cell released-EVs carrying surface PD-L1 were reported to evade immune surveillance by suppressing the function of CD8 T cells in melanoma (44). Also, melanoma cell-derived EVs induced the expression of PD-L1 on naïve myeloid cells converting them to myeloid derived suppressor cells via TLR signaling in melanoma mouse models (45). In CRC, TEVs were reported to promote metastasis by inducing macrophage activation via stimulating NOD1 (Nucleotide-binding oligomerization domain-containing protein 1) signaling. This EV-mediated release of inflammatory cytokines and chemokines from activated macrophages was found to promote liver metastasis of CRC (46). While Ludwig et al., 2022 showed the role of TEVs in promoting angiogenesis in HNSCC, by inducing a pro-angiogenic phenotype in the naïve macrophages and promoting their infiltration to the tumor site (47). Thus, these studies provide a rationale for immune evasion/suppression and development of therapeutic resistance in cancers, and indicate EVs as one of the important determinants driving clinical resistance.

Another important function of TEVs is the activation of fibroblasts to cancer associated fibroblasts (CAFs), which in turn stimulate the surrounding normal and tumor cells to acquire cancer-supporting properties, recently shown in ovarian cancer model (48). Also, EVs from pancreatic ductal adenocarcinoma (PDAC) cells were able to regulate the conversion of pancreatic stellate cells (PSCs) to CAFs by upregulating the expression of matrix metalloproteinase-9 (MMP-9) (49).

TEVs promote cancer metastasis by regulating the secondary organ niche, like prostate cancer derived EVs were shown to induce a pro-metastatic environment in the bone by stimulating osteoclastogenesis and bone resorption thereby promoting prostate cancer metastasis to the bone (50, 51). Furthermore, Ma et al., 2021 identified microRNA, miR-152-3p in the cargo of the prostate cancer-EVs that was found to promote osteoclastogenesis by inhibiting the expression of Mafb - a negative regulator of osteoclastogenesis. Similarly, Urabe et al., 2023 showed TEVs-mediated function in inducing a bone metastatic niche by osteoclast formation via CUB-domain containing protein induced NF-kB mediated signaling. Correspondingly, we identified the role of TEVs in promoting metastasis in melanoma (52) and NB (15) models by inducing the formation of pre-metastatic niche at potential metastatic sites like lung and liver respectively. Several other studies have demonstrated the role of TEVs in inducing PMN via different mechanisms in multiple cancer models (53–56). Breast cancer cell-derived EVs induced PMN by suppressing the glucose uptake of the niche resident non-tumor cells, thereby increasing nutrient availability for incoming tumor cells and facilitating their growth in breast cancer model (54). CRC-EVs were shown to affect the hepatic stellate cells, transforming them to CAFs, that induced the recruitment of myeloid derived suppressor cells to the livers, thereby inducing an immunosuppressive PMN (56). While PDAC-EVs were found to induce PMN in the liver by sequentially affecting the Kupffer cells, hepatic stellate cells, ultimately resulting in the recruitment of bone marrow derived macrophages and neutrophils (53).

Increased levels of TEVs detected in the bodily fluids of cancer patients and their association with tumor progression, further indicates the diagnostic/prognostic potential of EVs in different cancer types. Several studies have identified key tumor-specific antigens and nucleic acids in TEVs, and have demonstrated various methods for detecting and analyzing EV markers from cancer patient samples (57–63). Moreover, studies have identified specific markers and EV-signatures associated with metastatic cancer (60), thus suggesting the significance of TEVs to distinguish patients with advanced-staged metastatic disease.

Interestingly, besides the TEV-mediated signaling, several recent studies have identified the role of stromal cell-derived EVs in supporting cancer progression by affecting the tumor cells, thus indicating a bidirectional EV-mediated crosstalk in cancer. EVs derived from adipocytes and adipose-endothelial cells were found to induce proliferation, migration and invasion of the recipient prostate cancer cells, thereby promoting metastasis in prostate cancer (64, 65). While CAFs were found to support the surrounding tumor cells by transferring tumor-supportive signals via EVs, thereby contributing to tumor growth and metastasis in several cancer types (62, 66–68). In addition, endothelial cell derived-EVs were shown to affect macrophages, by converting them to a M2-like phenotype thereby inducing an immunosuppressive environment and aiding in cancer progression (69). Accordingly, TAMs-released EVs were shown to promote EMT and angiogenesis by regulating the tumor cells and endothelial cells, thereby promoting metastasis in hepatic cancer (70). Several other studies have reported EV-mediated transmittance of pro-tumor signals between TAMs and the recipient tumor cells in multiple cancer types (71, 72). These findings thus demonstrate an orchestrated EV-mediated network in cancer, which is although initiated by tumor cells but is contributed by several other tumor-supportive cells in the TME. EV-mediated signaling is thus a characteristic feature of majority of solid tumors that essentially regulates multiple steps during cancer progression, hence a better understanding of these diverse EV-regulated mechanisms is crucial for the development of novel, more efficient therapies for cancer.

## 3 Role of EVs in NB progression

### 3.1 NB-derived EVs in tumor growth, metastasis, PMN and microenvironment regulation

Since NB is a highly metastatic cancer with distant metastasis prominently to liver, bone and bone marrow (6), it is essential to enhance the understanding of mechanisms underlying the metastatic cascade in NB, in order to prevent NB metastasis. It is well-known that cancer cells exploit the supporting cells at primary site for survival, growth and increasing their metastatic potential, and that the TME plays a major role in regulating and supporting the early events of metastasis. Besides the tumor-stromal cell interactions at the primary site, tumor released factors like EVs play a major role in PMN at the future metastatic organs, thereby promoting the colonization of the incoming tumor cells and forming metastasis. Similarly, understanding of NB TME has progressed in the past years with an increasing focus on immune cells (like tumor-associated macrophages (TAMs), neutrophils), stromal cells, cancer associated fibroblasts (CAFs), and importantly the contribution of EVs in regulating the crosstalk between these stromal/immune cells, and with NB tumor cells.

Haug et al., 2015 and Marimpietri et al., 2013 first reported the presence of EVs in NB, and detected the EV-cargo potentially involved in tumor progression. They found the enrichment of oncogenic miRNAs along with the donor NB cell specific proteins in the isolated EVs (73, 74). Proteomic analysis of the EV-cargo further identified the presence of EV-markers, validating the endosomal-origin of these vesicles. Apart from the characteristic EV-biomarkers, other major proteins detected in the NB-EVs included fibronectin, cancer stem cell marker - prominin-1, immunosuppressive factor - B7-H3, and the ECM regulator – basigin, thus suggesting the potential of EVs in regulating the TME during NB progression (74). Several other studies have detected the presence of oncoproteins in the NB-released EVs indicating the importance of EVs as mediators of oncogenic signals and promoting cancer progression. Tsakaneli et al., 2021 demonstrated NB vesicle induced regulation of TME, by spreading oncogenic signals to non-MYCN amplified NB cells and stromal cells like tumor associated macrophages (TAMs) and CAFs. EVs secreted from MYCN-amplified NB tumors or cells were enriched in oncogenic glycolytic enzymes like pyruvate kinase M2 (PKM2) and hexokinase 2, and induced glycolysis and proliferation of the recipient cells. Cancer cells have known to mainly rely on aerobic glycolysis for sustained growth and metastasis, and specifically to adapt to new environment at secondary locations. In MYCN-amplified NBs, MYCN was found to transfer such aggressive, oncogenic phenotype to other non-MYCN amplified NB cells and surrounding stromal cells by regulating the host cell EV-protein cargo thereby promoting tumor progression (75).

Importantly, Colletti et al., 2017 showed that EVs derived from different NB cell lines obtained from either primary or metastatic tumors, released EVs with different proteomic profiles. Specifically, EVs released from primary tumor NB cell lines consisted of proteins involved in ECM regulation and adhesion, thus indicating the role of primary tumor released-EVs in inducing a pro-metastatic environment and promoting metastasis – a prominent function of TEVs. On the other hand, bone-metastasized cell line-derived EVs exhibited proteins associated with high mitochondrial activity, reflecting the ability of these cells to adapt their metabolism to a new environment, and which is potentially further transferred via EVs to other NB cells (76). Thus, the different and unique signatures of EVs specific to different stages of tumor progression reflects the versatile functions of EVs, and also suggests their potential use as biomarkers for NB-stage determination.

A recent study (77) reported the paracrine function of NB-EVs under hypoxic conditions, by imparting the pro-metastatic and aggressive phenotype to the surrounding normoxic NB cells. Hypoxia is a commonly observed event in solid cancers including NB, and is associated with increased neovascularization and metastasis. Previous studies have shown that hypoxia induces the secretion of hypoxia-inducible factors (HIFs) from NB cells exposed to low oxygen environment, that regulate angiogenesis, ECM remodeling, invasion, metastasis and resistance to chemo-, radiotherapy. Likewise, Fusco et al., 2023 identified the role of EVs in mediating the hypoxia-induced signaling in NB, and showed that NB cells release increased amounts of EVs when under hypoxia as compared to cells grown in normoxic environment. EVs derived from the hypoxic-NB cells exhibited a differential miRNA cargo that promoted the migration and invasion of the recipient normoxic NB cells in culture. Specifically, hypoxic-NB cell derived EVs were enriched in microRNA – miR-210-3p, which was transferred to the recipient target cells and imparted the hypoxia-induced aggressive phenotype to the recipient cells. Hypoxic condition led to the overexpression of miR-210-3p in NB cells that ultimately increased the levels of this miRNA in the secreted EVs. Moreover, EVs from re-oxygenated cells following hypoxia retained this ability of imparting aggressiveness to naïve NB cells, indicative of the possibility that cancer cells retain the memory of hypoxic environment and the conserved hypoxia-induced signaling via EVs (77).

Moreover, emerging studies have demonstrated the tumor-stromal cell crosstalk by EVs through directly interacting and reprogramming the stromal/immune cells. NB-EVs have been found to trigger the polarization of mesenchymal stromal cells (MSCs) to a pro-tumorigenic phenotype in the bone marrow niche. NB cell-derived EVs were taken up by the bone marrow derived MSCs, that stimulated the production of pro-tumor cytokines and chemokines (IL-6, IL-8, VEGF and monocyte-chemotactic protein-1) in the recipient MSCs, thereby inducing an inflammatory reaction that can potentially create a favorable microenvironment for BM metastasis (78). Correspondingly, another study showed the involvement of NB-EVs in regulating the MSCs (79). BM metastasis-derived-NB cell-EVs induced osteogenic differentiation in the BM MSCs, that can potentially contribute to creating a metastasis favorable environment and promoting NB metastasis in the BM. Of note these BM-metastatic NB cell-derived EVs exhibit a unique organ-specific miRNA profile with upregulated levels of miR-375, that was transferred to the MSCs and was responsible for osteogenic induction (79).

EV-miRNAs have recently been reported to regulate fibroblasts in the NB TME (80). Intracellular miR-574-5p was found to induce the synthesis of PGE2 in NB cells, which in turn was responsible to sort the miR-574-5p in the secreted EVs. These miR-574-5p enriched NB-EVs were found to stimulate the differentiation of fibroblast to CAFs, by inducing alpha-SMA expression via TLR7/8 signaling. CAFs have known to be one of the major components of the TME that mediate multiple cancer-promoting functions, importantly remodeling of the extracellular matrix (ECM) thereby promoting local invasion and distant metastasis (81, 82). Moreover, TEVs have found to activate normal fibroblasts to CAFs in several cancer models (20, 48, 83). Liver resident fibroblasts were found to take up primary NB tumor derived-EVs preceding metastasis (84), however the exact mechanisms behind EV induced CAF activation in NB are unclear. Similarly, our recent findings demonstrated the effect of NB cell derived-EVs on fibroblasts using an *in vitro* system (15). We showed that treatments with EVs derived from our highly metastatic M1 (NB) cells (15, 85) imparted invasive phenotype to the mouse fibroblasts, suggesting the EV-mediated CAF activation in NB, and a potential mechanism promoting NB metastasis.

One of the prominent features of TEVs is to induce PMN formation at the potential secondary locations, thus priming these organs for future metastasis (19, 20). Studies have shown the involvement of TEVs in the initiation and regulation of sequential events of PMN formation in multiple cancer models, thus promoting cancer-cell homing and growth at these new locations (15, 19, 53, 55). Besides, TEVs have a specific organotropism that reflects the preferred metastatic locations of the host tumor cells (87). Our study determined the significance of EVs in NB metastasis by inducing PMN formation at the potential metastatic sites (15). We identified IGF2BP1- an RNA binding protein as a promoter of metastasis in NB, and uncovered its pro-metastatic function that is mediated by EVs. Mechanistically, we found that IGF2BP1 increases the expression of its newly identified target- SEMA3A by stabilizing its mRNA, thereby upregulating its protein levels in NB cells and eventually in the secreted NB-EVs. In turn EV-associated SEMA3A was found to induce pre-metastatic niche (PMN) formation as evidenced by increasing fibronectin deposition and CD45^+^ cell accumulation, specifically at the preferred secondary site-liver. Fibronectin expression is a known marker of PMN, it regulates the remodeling of the ECM of the distant organs, thereby increasing the recruitment of myeloid-derived suppressor cells, generating a tumor-supportive environment and thus facilitating adhesion of the circulating tumor cells (17–19). Targeting IGF2BP1 or IGF2BP1-modulated SEMA3A expression in NB cells and their resulting EVs prevented this PMN formation and metastasis in mice, suggesting the potential therapeutic application of targeting this axis for NB treatments. Likewise, another study showed the importance of TEVs in PMN formation in NB, by demonstrating the capture of TEVs by the microenvironment cells, mostly macrophages and resident fibroblasts, at the potential metastatic organs (84). Uptake of TEVs by resident macrophages was found to induce the expression of inflammatory genes in the recipient cells as an event of the PMN, prior to the homing of cancer cells. Using tagged cells consisting of a GFP reporter anchored to the TEV membrane, this study captured the uptake of endogenous TEVs by the recipient macrophages in the microenvironment of specifically the future metastatic organs during the PMN event (84). TEVs are furthermore known to carry immunoregulatory proteins (like PD-L1, FasL), and have found to affect immune cell directed therapies in multiple cancer models. Ali et al., 2020 showed that NB cell derived-EVs specifically abrogated the cytotoxic function of (CD171-specific) CD4^+^ CAR T-cells without affecting their viability *in vitro*. These (L1 cell adhesion molecule or CD171) CD171-specific CAR T-cells target the CD171-antigen overexpressed on NB cells, and are currently being investigated under phase 1 trial (NCT02311621) for the treatment of recurrent/refractory NBs (88). TEVs thus should be considered for designing efficient CAR T cell-based therapies for NBs as well as other cancers.

**Figure 1:**
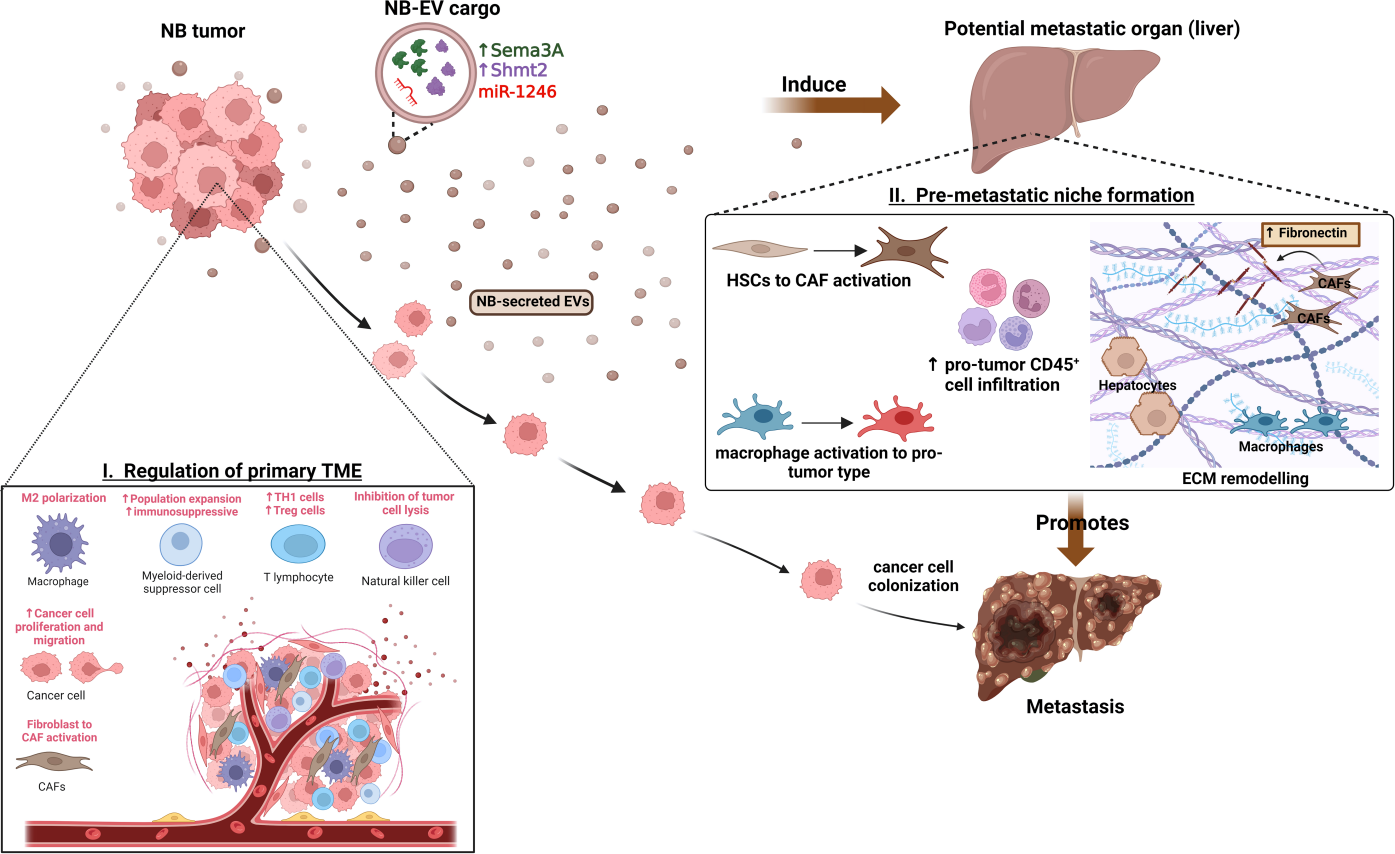
Functions of NB-EVs in TME and PMN regulation. I) NB-derived EVs regulate the primary tumor microenvironment by promoting M2-like macrophage polarization, recruitment and expansion of immunosuppressive cells, CAF activation and promoting cancer cell proliferation, migration. II) Specific cargo of NB-EVs (15, 84) have found to induce the pre-metastatic niche at potential secondary organs (like liver) by regulating the ECM remodeling, inducing immunosuppressive cell infiltration, and mediating the activation of CAFs and tumor-supporting macrophages, thereby promoting metastasis.

Thus, with the recent number of findings demonstrating the regulatory functions of TEVs on NB TME and in regulating the distant site tissue ME (**Figure 1** and **Tables 1**, **2**), EV-mediated mechanisms/pathways could serve as potential therapeutic targets for developing more efficient therapies for treating HR-NBs. While the potential of EVs or specific EV-cargo to directly affect the immune cells, and inducing an immunosuppressive environment could be further investigated for developing potential targets to inhibit this immunoregulatory and tumor-promoting effects of EVs.

**Table 1:** Functions of NB-EVs in NB - effects in culture.

Source of EVs	EV Function	Cargo	Recipient cells/organs	Proposed Mechanism	Reference
MYCN amplified NB cells (Kelly and SK-N-BE(2)-C)	Predicted functions - promoting cell growth, survival, movement processes.	miRNAs (mir-16, 125b, 21, 23a, 24, 25, 27b, 218, 320a, 320b and 92a	HEK-293T, SK-N-AS, HUVEC	**_**	Haug BH et al. (73),
NB cell lines - HTLA-230, IMR-32, SH-SY5Y and GI-LI-N	Predicted functions - increasing defense response, cell differentiation, cell proliferation, immune regulation, ECM remodeling.	fibronectin, prominin-1, B7-H3, basigin	**_**	**_**	Marimpietri D et al. (74),
MYCN upregulated neuroblastoma cell line - TET21-N	Increase cell proliferation, metabolic activity by inducing glycolysis.	pyruvate kinase M2 (PKM2) and hexokinase 2	NB cell lines - SH-SY5Y, SH-EP (stromal-type phenotype)	EV-PKM2 induces histone H3 phosphorylation, thereby activating c-MYC expression in recipient cells.	Tsakaneli A et al. (75),
Established NB cell lines (IMR32, SK-N-SH, SH-SY5Y, SKNBe2c and LAN-1); patient derived cell lines (IGR-NB8, IGR-N91 and CHLA255)	Predicted function- increasing ECM regulation and adhesion, mitochondrial activity, cell survival, proliferation and cancer progression.	Differential EV-Protein cargo: specific to primary tumors - dystrophin (DMD), TNFAIP3-interacting protein (TNIP1), ELAV-like protein 2 (ELAVL2), Nova-2 (NOVA2), E3 ubiquitin-protein ligase pellino homolog 2 (PELI2); and specific to bone-marrow metastasis - Signal peptidase complex catalytic subunit SEC11 (SEC11A), Nuclear pore complex protein Nup107 (NUP107), Calcium and integrin-binding protein 1 (CIB1)	**_**	**_**	Colletti M et al. (76),
NB cell lines - SK-N-AS and SK-N-DZ (hypoxia conditioned)	Increase cell migration, invasion and colony formation.	miR-210-3p	SK-N-AS and SK-N-DZ	miR-210-3p mediated regulation of target mRNAs, possibly like E2F3, TNIP1, SOCS1, FGFRL1, and STAT3.	Fusco P et al. (77),
NB cell lines- SK-N-BE (2), CHLA-255 and NB-19	Inducing a pro-metastatic environment, by polarizing MSCs towards a protumorigenic phenotype.	Gal-3BP (Galectin-3 binding protein)	bone-marrow mesenchymal stromal cells (MSCs)	EV-uptake induces chemokine/cytokine production via ERK1/2 and AKT activation in MSCs.	Nakata R et al. (78),
NB cell lines - SK-N-AS and SK-N-SH	Regulation of tumor microenvironment (TME), by promoting the differentiation of fibroblast to CAFs.	miR-574-5p	human fetal lung fibroblast cell line HFL1	miR-574-5p - CUG binding protein 1 interaction induces PGE2 biosynthesis in NB cells, leading to miR-574-5p sorting in EVs. miR-574-5p enriched EVs activate TLR7/8 signaling in the recipient fibroblasts.	Proestler E et al. (80),
NB cell line - SH-SY5Y	Hinder immune-cell mediated therapy.	**_**	healthy donor derived CD4+ and CD8+ T cells, transduced for CAR expression.	Inhibits CD4+ CD171-specific CAR T cell cytotoxic function.	Ali S et al. (88),
NB cell line - SK-N-BE2 (MYCN amplified)	Impart chemoresistance (towards doxorubicin).	Protein cargo - TSG101, FLOT1, VPS35, NEDD4, B-catenin and RHOA	SH-SY5Y (non-MYCN amplified)	Promoted cell growth, migration; and imparted protection towards doxorubicin treatment to the recipient cells.	Fonseka P et al. (89),
Senescence induced IMR32 cells	Impart resistance towards therapy-induced-senescence.	miR-378b and miR-205-5p	**_**	Predicted mechanism - cellular metabolism regulation.	Zhou X et al. (90),
NB cell lines - SH-SY5Y, SK-N-BE (Irradiated)	Impart radio-resistance, induce survival, proliferation and migration.	**_**	non-irradiated SH-SY5Y and SK-N-BE cells	Potentially upregulating DNA damage repair pathways pATM, BRCA1, and p53 in the recipient cells.	Tortolici F et al. (91),
NB cell lines - SK-N-BE (2), CHLA-255 and IMR-32	Impart chemoresistance (towards cisplatin).	miR-21	human monocytes	NB cell derived EV-miR-21 activates TLR8-NF-кB signaling in monocytes, resulting in miR-155-enriched EVs. Monocyte derived EV-miR-155 regulates TERF1 in NB cells.	Challagundla KB et al. (92),

**Table 2:** Function of NB-EVs in NB progression - *in vivo* effects.

Source of EVs	EV Function	Cargo	Recipient cells/organs	Proposed Mechanism	Reference
NB cell lines - SKNSH, SHSY5Y, IGR-N91, IGR-NB8, IMR32, SKNBe2 c and LAN1; and NB patient plasma derived EVs	Inducing pro-metastatic environment, by promoting osteogenic differentiation.	miR-375	healthy donor derived bone-marrow MSCs; *in vivo* - mouse bones	Target prediction analysis reveal potential miR-375 targets - Yes-Associated Protein 1 (YAP1) and DEP domain containing mTOR-interacting protein (DEPTOR).	Colletti M et al. (79),
Murine NB cell lines - M1 and 9464D	Induce the formation of pre-metastatic niche (PMN) in livers, and promote NB metastasis.	SEMA3A and SHMT2 proteins	*in vivo* - mouse livers; *in vitro* - NIH3T3 mouse fibroblast cells	Induces PMN formation by increasing fibronectin expression and CD45+ cell accumulation in the liver.	Dhamdhere MR et al. (85),
NB cell lines - CHLA-136 and SK-N-BE (2)	Induce the formation of PMN in liver.	miR-1246	murine RAW 264.7 macrophage cell line; *in vivo* - mouse liver macrophages, hepatic stellate cells and hepatocytes	Induces the expression of inflammatory genes in the recipient macrophages and hepatic stellate cells.	Blavier L et al. (84),
SK-N-AS and murine NB cell line - 9464D	Impart resistance towards doxorubicin, and promoted metastasis.	PTX3 and PLAT proteins	*in vivo* - mouse lungs, bone and liver tissues.	Doxorubicin treatment increases the secretion of EVs from NB cells and differentially regulates the EV-cargo, the altered EV-cargo induces PMN in mice.	Wills CA et al. (86),
NB cell lines - IMR32 (human), 9464D (murine)	Impart resistance towards GD2-immunotherapy (dinutuximab).	**_**	*in vivo* - mouse lungs, liver, spleen; human established NK92 cell line	NB-EVs inhibit the infiltration of effector NK-cells, and increase the population of TAMs, thereby promoting an immunosuppressive microenvironment and conferring resistance towards dinutuximab.	Liu X et al. (93),

### 3.2 NB-EVs in therapeutic resistance

Despite significant medical advances in treatment, therapeutic resistance is a major reason for treatment failure in NB. Understanding the mechanisms of therapy resistance is thus essential to develop reversal strategies and improve disease prognosis in NB. Cancer cells either exhibit primary resistance towards drugs or develop characteristics of resistance after long-term chemotherapy (acquired resistance). Inter and intra-tumor genetic heterogeneity (mutations, amplification), epigenetic changes (DNA hypermethylation, histone modifications), and several mechanisms like aberrant expression of miRNAs, overexpression of drug efflux transporters, cancer cell stemness, autophagy, anti-GD2 antibody internalization and TME interactions are responsible for therapy resistance in NB cells (94). One of the most direct ways for tumor cells to develop resistance is via physical means by restricting access of the drug to target site, by increasing the expression of ATP-binding cassette (ABC) transporter (efflux transporter) proteins. Over expression of these efflux transporter proteins have been associated with resistance to commonly used chemotherapeutic drugs like vinblastine, doxorubicin, vincristine and paclitaxel. Major ATP driven efflux transporters like P-gp or MDR1 (P-glycoprotein), BCRP (breast cancer resistance protein), and MRP1 (multidrug resistance protein 1) are responsible for chemoresistance *in vivo* (95). Importantly, MRP1 has been shown to regulate tumor response to chemotherapy in MYCN amplified NBs (96), and several other ABC transporters like ABCC3 and ABCC4 that are transcriptionally regulated by MYCN were found to be associated with poor disease prognosis in NB (97).

Abnormal expression of certain miRNAs has been reported as another major contributing factor of therapeutic resistance in cancers (94, 98). Several miRNAs were associated with drug resistance in NB by imparting chemoresistance phenotype to NB cells (94, 99). MiR-17-5p was found to render drug resistance in NB by regulating P21 and BIM genes thereby promoting cell proliferation and therapeutic resistance (100, 101). MiR-21 was found to be upregulated in cisplatin-resistant NB cells and its ectopic expression in parental cells resulted in increased drug resistance and proliferation (102, 103). While MiR-137 is negatively correlated with drug resistance via regulating the expression of CAR (constitute androstane receptor) in doxorubicin-resistant NB cells (104). Since EVs are known to carry an array of cargo consisting of proteins, non-coding RNAs including miRNAs (105, 106), these miRNAs may be transferred via resistant NB cell derived-EVs to the recipient less aggressive NB cells, thus imparting them the drug-resistant phenotype.

Also, it is well-known that inflammatory and immunosuppressive TME is involved in tumor resistance (107), and EVs – an important component of the TME have found to regulate therapeutic resistance via direct or indirect signaling with the stromal/immune cells in several cancers (92, 93). Challagundla et al., 2015 showed the EV-dependent crosstalk between NB cells and monocytes conferring cisplatin resistance to NB cells. NB cell derived EV-miR-21 induced TLR8/NF-kB signaling in monocytes/macrophages that resulted in miR-155 secretion via EVs. Macrophage derived EV-miR155 in turn directly targeted TERF1 (telomerase inhibitor) in NB cells, leading to telomerase lengthening and activation, thereby imparting cisplatin-resistance to NB cells. Furthermore, use of EV-secretion inhibitor (GW4869) (108) restored NB cell sensitivity to chemotherapeutics, thus indicating the potential of using (exosome) EV inhibitors to prevent drug-resistance or as a reversal strategy.

MYCN amplification is a known marker of tumor progression and resistance in NB (109), and EVs derived from MYCN-amplified NB (SK-N-BE2) cells were found to impart doxorubicin-resistance to non-MYCN amplified recipient SH-SY5Y cells (89). Moreover, MYCN-amplified-cell-derived-EVs were reported to possess a differentially regulated protein cargo, with enrichment of proteins (like TSG101, FLOT1, VPS35, NEDD4, β-catenin and RHOA) that were associated with signal transduction, induced growth and migration. Incubation of recipient SH-SY5Y cells with such MYCN-amplified-cell-derived EVs transferred cell growth, migration ability and protection towards doxorubicin treatment to these sensitive cells. Whereas, EVs from MYCN-knock down SK-N-BE cells failed to impart protection against doxorubicin to the recipient SH-SY5Y cells, thus implicating the importance of EVs in transferring aggressive phenotype to the neighboring sensitive cells, and their potential in mediating the interaction between different clonal subpopulations within the heterogenous tumor.

Similarly, doxorubicin treatment was found to accelerate EV-secretion from NB cells and differentially regulated the protein cargo of the released EVs. These doxorubicin-treated NB cell-derived EVs were enriched in metastasis and chemoresistance promoting proteins (PTX3 and PLAT), and accelerated metastasis in NB mouse models by inducing the formation of pre-metastasis niche (86).

Another study (90) demonstrated the role of TEVs in escaping therapy-induced-senescence in human NB cells, and imparting this phenotype to the neighboring cancer cells by transfer of certain miRNAs. The differentially expressed miRNAs identified in the EVs released from AURKA-inhibitor treated senescent cells were associated with metabolism regulation, suggesting that cells package these miRNAs in EVs as signals in response to senescence pressures.

Additionally, EVs have also shown to transfer radiotherapy-induced pro-oncogenic signals from irradiated cells to non-irradiated cells in NB (91). Although irradiation (IR) is majorly included in the multi-modal therapeutic regimen for treating advanced NBs, the use of IR has been associated with increased risk of developing secondary neoplasms. Irradiations (IRs) have been shown to trigger epithelial to mesenchymal transition (EMT), increase cancer cell dissemination, invasion, adhesion and angiogenesis thereby promoting metastasis and tumor aggression (110). Importantly, multiple studies showed that cells not directly treated with IRs accumulated genetic mutations and epigenetic aberrations, that were mediated via EVs secreted by irradiated cells. Tortolici et al., 2021 showed that IR exposure increased the secretion of EVs from SH-SY5Y NB cells, and that these EVs consisted of a differential protein cargo. Moreover, these IR-exposed-cell derived EVs were found to be efficiently taken up by the non-irradiated NB cells, and were found to induce survival signals, proliferation, migration and imparted radio-resistance to the naive recipient NB cells.

The most recent anti-GD2 immunotherapy by itself or together with immunostimulatory agents has substantially improved survival in patients with high-risk NBs (111, 112). Anti-GD2 antibody binds the cell surface disialoganglioside (GD2) antigen, that is expressed by the tumors of the neuroectodermal origin including NBs. NB tumor bound anti-GD2 antibody recruits immune effector cells like neutrophils and natural killer (NK) cells that trigger anti-tumor functions by antibody-dependent cellular cytotoxicity (ADCC) or complement-dependent cytotoxicity (CDC).

Additionally, incorporation of anti-GD2 antibody (dinutuximab) to post-consolidation regimen has also improved patient outcomes in refractory or relapsed groups (113). However, despite of this, more than 40% of patients fail to respond and develop resistance to anti-GD2 immunotherapy (7). Loss of GD2 expression on NB cells and antibody internalization by target NB cells have been found to be the major mechanisms of immunotherapy escape in NB (114). Several studies have indicated the importance of TEVs in immunotherapy resistance by interacting and suppressing the effector immune cells (like T-cells and NK cells), thereby modulating the host immune response in multiple cancer models (115–118). Furthermore, TEVs have found to carry cargo that reprogram the immune cells and hamper the host immune system. Recently, Liu et al., 2022 demonstrated the role of NB-cell-derived EVs in imparting resistance to dinutuximab by regulating the immune cells systemically as well as in the TME. NB-EVs were found to suppress the infiltration of dinutuximab-induced NK cells and increased TAM localization in the tumors, thereby promoting tumor growth in mice. Moreover, NB-EVs also inhibited the dinutuximab-triggered mobilization and maturation of NK cells in the spleen. While treatment with Tipifarnib (inhibitor of EV biogenesis/secretion) enhanced the efficacy of dinutuximab, by inhibiting the EV-induced immune-suppressive phenotype, and reduced tumor growth in mice (93).

**Figure 2:**
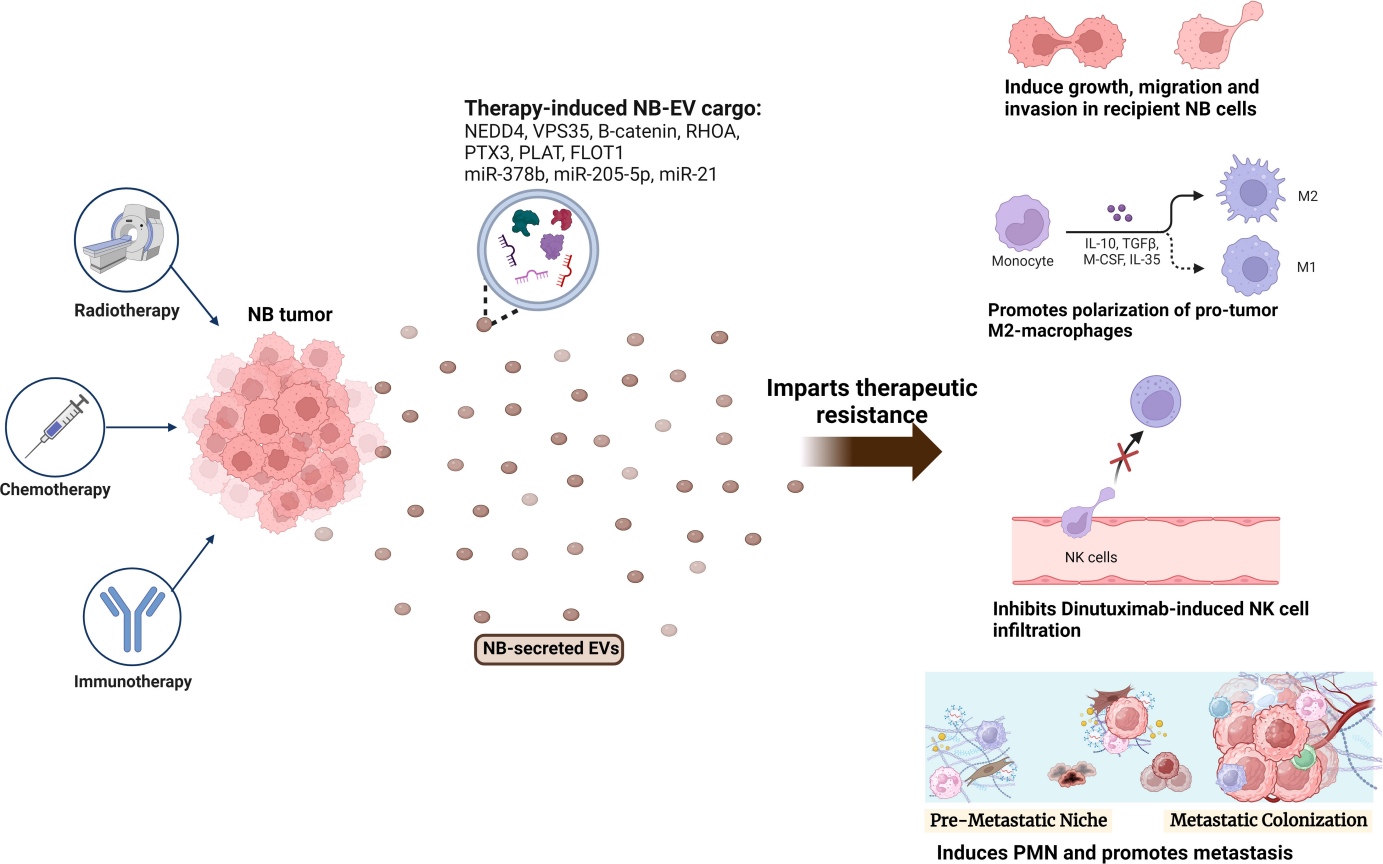
Role of NB-EVs in imparting therapeutic resistance. NB tumors treated with chemotherapy (doxorubicin, cisplatin), radiotherapy or immunotherapy (Dinutuximab) secrete EVs with a differentially regulated cargo, that imparts therapeutic resistance by regulating the sensitive recipient NB cells, and inducing an immunosuppressive microenvironment via promoting M2-like macrophage polarization, inhibiting NK-cell infiltration and promoting PMN formation.

Collectively, these findings suggest that EVs secreted from aggressive host NB cells mediate therapeutic resistance by transferring resistance signals to the sensitive recipient cancer (NB) cells or by reprogramming the TME and surrounding non-cancer cells (**Figure 2** and **Tables 1**, **2**), aiding in cancer progression and relapse. Hence targeting EVs-cargo or EVs-biogenesis/secretion could be an efficient novel approach for the treatment of radio-, chemo- or immunotherapy resistant NBs.

### 3.3 NB-EVs in NB diagnosis and prognosis

Current diagnostic and disease monitoring approaches for NB include x-rays, CT scan, MRI, screening for urine catecholamines like vanillylmandelic acid (VMA) and homovanillic acid (HVA), serum protein levels and tissue biopsies (119, 120). The initial diagnosis of NB involves laboratory procedures for testing full blood count, serum electrolyte levels, accessing liver function and abdominal ultrasound (121). Increased clinical resistance and higher disease relapse in patients with advanced-stage NBs result in poor prognosis and lower overall survival (20%) (7). The time from diagnosis to disease relapse is < 22 months, and the post relapse survival time is significantly low (4-5 months) (8, 122), thus providing a small therapeutic window to detect and treat these resistant NBs. Detection of minimal residual disease is essential to efficiently monitor the disease burden and could serve beneficial to achieve better prognosis.

Serum proteins like lactate dehydrogenase (LDH), neuron-specific enolase (NSE) and ferritin are currently used biomarkers for diagnosis and prognosis. Serum LDH levels have found to rise with tumor burden, and high levels of LDH has been linked with poor prognosis, however independent of disease stage in MYCN amplified NBs (123, 124). Another study reported it as a predictor of poor disease-free and overall survival in patients with advanced-stage NBs and older than 18 years of age (125). Thus, with the diverse reports, serum LDH levels may be used as a prognostic marker for monitoring real-time tumor burden, however, may not serve as an efficient diagnostic marker and for early intervention of the disease. Neuron-specific enolase (NSE) is an isoform of the glycolytic enzyme enolase specific to neural tissues, and is found in high levels in the serum in different endocrine tumors like carcinoids, medullary thyroid carcinoma, pituitary adenomas, islet cell tumors, small-cell lung carcinoma, malignant pheochromocytoma, ganglioneuromas, schwannomas and NBs (126–132). A few studies have also reported increased serum levels of NSE in melanoma, renal cell carcinoma, merkel cell tumor and Guillain-Barre syndrome (127). Hence, as elevated NSE levels are not specific to NB, it may be used as a general prognostic marker for monitoring therapeutic response and disease progression in cancer patients, although not specifically for NB. NB tumors also secrete glycosylated form of ferritin (123), distinct from the non-glycosylated form that is produced by the healthy cells (133). However, elevated serum levels of ferritin are found in stage 3 as well as more aggressive stage 4 NBs (120, 134, 135), thus may not be an efficient marker independently for diagnosis and determining the stage.

NBs secrete urine catecholamines such as vanillylmandelic acid (VMA) and homovanillic acid (HVA), and elevated levels of these metabolites have been used as noninvasive diagnostic markers in NB (123, 136–138). Low VMA to HVA ratio is associated with undifferentiated tumors and poor prognosis (123, 137). However, their diagnostic sensitivity is not optimal (137, 138). Moreover, the recommended 24-hour sample collections for metabolite measurements have been found to be challenging for the young patients that require the use of catheter or urine collection bags. VMA/HVA screening trials have demonstrated that there was no improvement in the NB specific mortalities among the screened patients (139, 140). While a few other retrospective studies have evidenced overdiagnosis of the favorable, spontaneously-regressive stage 1 tumors (120, 141–143).

Recently, targeted therapy is an evolving treatment for NBs, which currently utilizes tissue biopsy for identifying the genomic aberrations/signaling pathways. Prominent genetic alterations serve as beneficial markers, and have been routinely used for diagnosis, risk-classification and prognosis in NB (120). However, repeated tissue biopsies or surgical acquisition is not amenable for NB due to limitations like patient age, comorbidity, difficulty of obtaining adequate amount of tissue, time and costs, thus leading to clinical complications (120, 144)). Moreover, such tissue sampling is inefficient for comprehensive characterization of a tumor, since studies have demonstrated high intra-tumoral heterogeneity in primary/metastatic/relapsed tumors in several cancers including NB (145–147). Due to the dynamically changing genetic profile within the tumor over time, future treatment decisions made based on previous molecular information are thus inaccurate and less efficient.

Hence emerging studies have indicated the importance of liquid biopsies for better disease surveillance and developing cancer precision medicine (148, 149). Such minimally invasive tests include analyzing the body fluids (blood, spinal fluid, marrow) for cancer cell shed circulating free DNA (cfDNA) and RNA, disseminated circulating tumor cells (CTCs) themselves, and more recently tumor derived EVs (TEVs) (148–151). Higher levels of tumor cell released cell-free RNA detected in the plasma of patients with metastatic NBs (152), suggests the potential of circulating cell-free RNA as a biomarker for aggressive NB via liquid biopsy. Several techniques like qPCR, digital-PCR, RT-PCR, FISH, different sequencing methods and immunocytochemistry can be used to detect these nucleic acids and CTCs from liquid biopsy. Thus, liquid biopsies have overcome the limitations of conventional tissue biopsy, and can be utilized for repeated sampling thus capturing the genetic changes underlying tumor evolution in real-time (120, 148). Current technologies encompassing different biomarkers via liquid biopsies have been extensively reviewed elsewhere (148), we thus specifically focus on the potential of TEVs for NB diagnosis and prognosis in this review (**Tables 3**).

**Table 3:** Diagnostic/Prognostic potential of EVs in NB.

Source of EVs	Cargo	Reference
NB patient plasma	mutations in ALK, CHD5, SHANK2, TERT, FGFR1, PHOX2B and BRAF; genomic mutations in relapsed NB-EVs - ALK, TP53 and RAS/MAPK	Degli Esposti C et al. (153),
Plasma from patients on different chemotherapy, enrolled in HR-NBL SIOPEN study	differential miRNA signature between responders vs non-responders (miR-29c, miR-342-3p and let-7b)	Morini M et al. (154),
Plasma from NB patients and healthy control donors; NB cell lines (SK-N-SH, SH-SY5Y, SK-NBE (2)), HUVEC, HEK293, and human fibroblast cell line (MRC5)	miR199a-3p	Ma J et al. (155),
Plasma from patients newly diagnosed with NB (not yet on treatments); NB cell lines - SK-N-SH, SH-SY5Y and SK-N-BE	hsa-piR-1089	Wang H et al. (156),
Plasma from NB patients	NCAM, NCL, LUM and VASP proteins - NB disease markers; Myosin-9, fibronectin, calreticulin, AKAP12 and LTBP1 - markers for HR-NBs	Morini M et al. (157),

TEVs carry diverse cargo consisting of DNA, RNAs, non-coding RNAs, proteins and other biomolecules that reflect the donor cancer cell genomic/transcriptomic profiles (22, 158). Since cancer cells secrete substantial amounts of EVs which can be easily isolated from the body fluids (150, 154, 159), TEVs could be efficient futuristic biomarkers for NB. Correspondingly, NB-EVs were found to carry DNA that reflected the host cellular exome and carried tumor-specific genetic mutations. Such NB-EVs isolated from patient plasma consisted of genetic mutations in some of the known oncogenes and tumor suppressor genes in NB (like ALK, CHD5, SHANK2, TERT, FGFR1, PHOX2B and BRAF) (153). EVs from patients with relapsed NBs carried mutations of ALK, TP53 and RAS/MAPK genes that are linked with acquired resistance in NB (153). Thus, the unique genomic signatures of the EVs representing their host NB cells could serve as beneficial molecular markers for NB diagnosis and prognosis.

Recent studies focusing on EV-miRNAs have indicated their significance as non-invasive biomarkers in different malignant tumors (62). Similarly, several studies have reported the presence of small RNAs and their potential application for NB prognosis. Morini et al., 2019 demonstrated the potential of using EV-microRNAs as diagnostic markers and for predicting response to chemotherapy in NB patients. Importantly, this was the first study detecting the presence of significant amounts of NB-released EVs in the patient plasma at diagnosis, which reduced after chemotherapy thus suggesting the potential application of NB-EVs for disease monitoring. Moreover, the study identified differential miRNA profiles from the plasma derived EVs of responder vs non-responders to chemotherapy. Plasma derived EVs from children with HR-NBs (majority of which had stage 4 disease) under HR-NBL-1/SIOPEN treatment trial before and after induction chemotherapy were collected. A comparative analysis of EVs was performed to identify a miRNA signature discriminating good vs poor responders. Primary analysis identified a total of 62 significantly downregulated EV-microRNAs after induction chemotherapy. To further determine if the observed differential miRNA expression was associated with response to therapy, comparison was conducted between patients that showed (poor) minor response vs patients that had very good partial response (VGPR) towards chemotherapy. Three EV-microRNAs (miR-29c, miR-342-3p and let-7b) were significantly downregulated in patients with minor response, while their expression was retained in VGPR patients after treatment, showing the association of these miRNAs with better chemotherapy response. Two of the identified miRNAs (miR-29c and let-7b) are known regulators of MYCN, and miR-342-3p has previously shown to exert tumor suppressor functions in other cancer types. Thus, the determined EV-miRNA signature could be utilized early in time to identify patients resistant to the employed chemotherapy and need a modification in their therapeutic strategy, hence improving prognosis for NB. Similarly, Ma et al., 2019 identified miR199a-3p in the plasma derived EVs of NB patients and showed that its expression correlated with disease severity, thus suggesting the diagnostic/prognostic potential of this EV-miRNA via fast, easy and non-invasive detection method. Mechanistically, hsa-miR199a-3p was found to promote NB cell proliferation and migration by regulating its target NEDD4 gene expression (155).

Another type of non-coding RNA – the P-element induced wimpy testis (PIWI)-interacting RNAs (piRNAs) were also found in the plasma derived-EVs of NB patients. Significant levels of hsa-piR-1089 were detected in the EVs isolated from the plasma of NB patients, and had high sensitivity as well as specificity with the onset of NB. Furthermore, hsa-piR-1089 was highly expressed in the tumor tissues with metastatic disease, suggesting its potential as a prognostic marker. hsa-piR-1089 promoted tumor progression by downregulating the expression of KEAP1 – a tumor suppressor in NB. Few other studies in the past have demonstrated the role of piRNAs in tumor development, like piRNA-39980 was found to promote tumor progression and impart chemoresistance in NB cells. Thus, this new class of small non-coding RNAs could be used for early diagnosis and to provide a valuable reference for prognostic risk assessment in NB (156).

Similarly, TEVs also carry specific protein cargo that function as signal mediators in regulating the recipient cells/organs thereby promoting cancer progression. A recent study (157) reported that plasma derived EVs from NB patients showed a different proteomic cargo relative to their healthy age-matched control subjects, thus suggesting the potential application of EVs in NB diagnosis and for initial disease intervention. Proteomic analysis of the EVs identified differential levels of NCAM, NCL, LUM and VASP proteins in the EVs derived from NB patients, relative to the control subject EVs. Of which, NCAM (neural cell adhesion molecule), NCL (nucleocin) that were upregulated in the NB-EVs are known to exert tumor promoting functions. While LUM (lumican), DCN (decorin) and VASP (vasodilator stimulated phosphoprotein), that are known tumor suppressors were found to be downregulated in the EVs derived from NB patients (157). Network analysis identified strong protein-protein interactions between these, thus indicating their potential biological connections and functional relevance. Top enriched pathways associated with NB-EV proteins included cancer progression processes such as immune system regulation, inflammation, stress response, cell motility and cytoskeletal rearrangements. The expression profiles of HR-NB-EVs and LR-NB-EVs were compared to further identify the risk-group associated discriminating biomarkers. Interestingly, distinct EV-proteome cargo profiles exclusive to HR-NB and LR-NB each were identified. Myosin-9, fibronectin and LTBP1 (latent-transforming growth factor-beta-binding protein 1) which all are known positive regulators of cell migration & metastasis were found to be enriched in HR-NB-EVs. While the downregulated proteins in the HR-NB EVs included antitumor function exerting proteins like Calreticulin and AKAP12 (A-kinase anchor protein 12). Consistently, pathway analysis determined that these HR-NB specific EV-proteins were not only associated with immune regulation and cell proliferation, but were also involved in regulating metastasis processes like migration, adhesion and angiogenesis, hence suggesting an important role of EVs in promoting metastasis in HR-NBs. The discriminating power of the identified EV-proteins was accessed by generating the receiver operating characteristic (ROC) curves and measuring the area under the curve. Data showed that these identified EV-proteins NCAM, NCL, LUM and VASP were of high diagnostic value, discriminating NB patients from the healthy controls, thus indicative of their potential as efficient and non-invasive diagnostic markers for NB. While the expression of Myosin-9, fibronectin, LTBP1, calreticulin and AKAP12 are effective in differentiating the HR-NB and LR-NB patients. Thus, such unique EV-proteome signatures could be highly beneficial tools in stratifying patients at risk of developing aggressive disease.

Moreover, a recent study (160) has established an EV-based workflow, utilizing MVs (microvesicles), to identify MYCN status in NB, by detecting MYCN mRNA in the plasma derived EVs. High copy number of MYCN gene results in increased levels of MYCN mRNA in the cells, that were enriched and detected specifically in the derived MVs. Hence, these large EVs have also been proposed as potential diagnostic markers in NB with their applicability for rapid detection of MYCN status unlike the conventional methods.

With the ease and accessibility of obtaining TEVs from patients by the means of liquid biopsies, these could become efficient tools for NB diagnosis, and disease monitoring that requires repeated sampling. Besides, TEV-cargo could be informative tools for accessing the dynamic and heterogeneous genetic and transcriptomic profiles of NB tumors, which could be of high beneficial value for designing personalized therapeutic strategies for treating resistant/relapsed NBs. Even though not yet ready to be translated to clinic, with the current advances in EV-detection and profiling technologies (161), patient-derived TEVs may serve as a highly resourceful tool for detecting minimal residual disease, thus preventing recurrent NBs.

## 4 Therapeutic potential of targeting EVs for NB treatment

**Figure 3:**
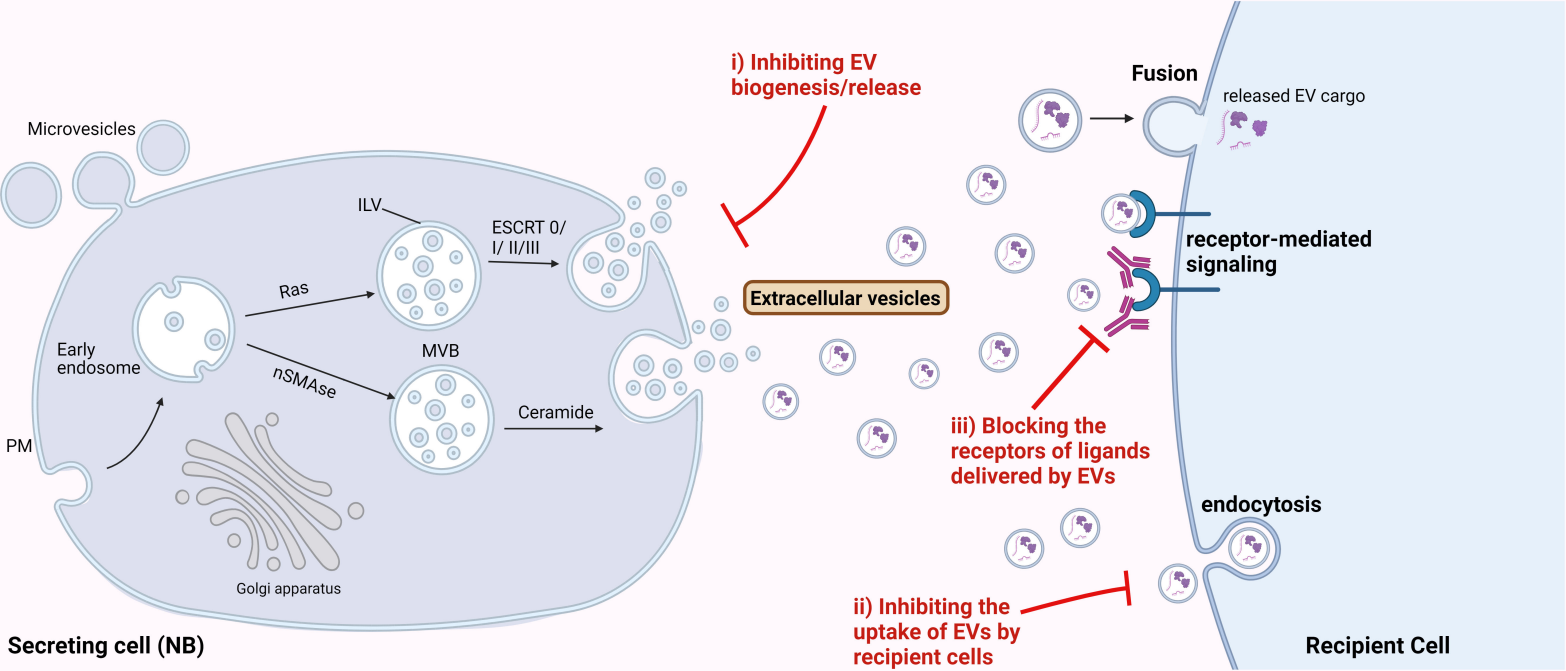
Therapeutic potential of targeting EVs for NB treatment. Possible strategies to target EVs for NB treatment could be by i) inhibiting the biosynthesis or release of EVs from NB cells, ii) inhibiting the uptake of NB-derived EVs by the recipient cells, and iii) blocking the receptors for the ligands delivered by EVs, thereby inhibiting the receptor-mediated signaling in the recipient cells.

As described in various sections above, TEVs are important regulators of NB progression, thus could be efficient targets for developing novel therapies for NB treatment. The major EV-associated strategies could include: *i)* targeting the biosynthesis or release of EVs, *ii)* blocking the uptake of NB-EVs by the recipient cells, and *iii)* blocking the receptor-mediated signaling induced by EV-cargo in the recipient cells (**Figure 3**). Several *in vitro* studies utilizing different cancer cell lines have indicated the significance of different EV-release blockers in inhibiting EV secretion (162, 163). Some of the well-known compounds specifically inhibiting the formation and secretion of EVs include GW4869 and manumycin A. GW4869 inhibits the membrane neutral sphingomyelinase (nSMase), thus preventing the formation of intraluminal vesicles and resulting EVs. nSMase is essential for intra-luminal vesicle formation via the ESCRT-independent pathway, by converting sphingomyelin to ceramide. While manumycin A targets the ESCRT-dependent pathway thereby preventing the formation of multivesicular bodies, and blocking EV biosynthesis and release (162). Several other EV biogenesis and release inhibitors are being tested for their potential therapeutic application for different cancer types (163). But substantial amount of pre-clinical studies demonstrating the efficacy of these compounds are lacking. A recent breast cancer study showed that these compounds were able to significantly (65-98%) reduce EV release from multiple triple-negative breast cancer cell lines (164) thus, suggesting the potential of inhibiting EV-release for preventing NB progression. Likewise, Liu et al., 2022 utilized tipifarnib - a farnesyltransferase inhibitor, that is known to specifically target the release of EVs, to inhibit EV-secretion in NB mouse model. Inhibition of EVs was found to enhance the efficiency of the GD2-immunotherapy in mice, by rescuing its NK cell-recruitment function (93). These data suggest that inhibition of EV-release from NB cells could be an efficient therapeutic approach for preventing NB metastasis. However, the limitation of these inhibitors to specifically target TEVs needs to be addressed, since even healthy cells release EVs for physiological processes.

Furthermore, we determined IGF2BP1-SEMA3A as the regulatory axis, and importantly showed the effect of SEMA3A-EVs on recipient cells/organs *in vitro* and in mice. Thus, blocking the uptake of NB-EVs by the recipient cells could be another potential strategy for targeting EV-mediated effect in NB progression. EVs have known to bind the target cells via specific receptors, surface proteins, lipids and glycans (165), thus targeting or blocking these can be an efficient approach to inhibit the EV-target cell interactions. Next, after binding to the target cells, EVs affect the recipient cells via either of the uptake mechanisms such as endocytosis, fusion, or by direct receptor-mediated signaling (165). Thus, utilizing endocytosis inhibitors like chlorpromazine might prevent the NB-EV-recipient cell associations. Chlorpromazine was shown to inhibit the formation of clathrin-coated pits thereby reducing the EV-uptake in ovarian cancer (165).

Additionally, since EVs can trigger intracellular signaling in the recipient cells via direct interaction with the surface receptors, blocking the receptors on the recipient cells could be a potential strategy to inhibit the EV-mediated signaling induced by EV-cargo. For instance, the effect of EV-SEMA3A could be inhibited by blocking its receptor Nrp1 on the recipient cells, thereby preventing the formation of PMN, and potentially metastasis in NB.

## 5 Conclusions

In conclusion, NB is a highly metastatic cancer, with more than half of all NB patients diagnosed with high-risk (HR) distant metastatic disease. Despite treatments with recent multi-modal radio-chemo-immunotherapy, 5-year survival in the HR group still remains <50%, due to increased disease relapse. Moreover, NB tumors have found to be highly heterogenous and dynamic, and exhibit intra-tumoral genetic heterogeneity. Given the aggressive nature and complexity of this pediatric cancer, it becomes essential to better understand the biology and mechanisms underlying NB progression, specifically metastasis, and identify efficient biomarkers and novel therapeutic targets for treating metastatic and clinically resistant NBs. EVs have emerged as a promising area of investigation with their potential to be utilized as diagnostic/prognostic marker for NB, and as a target for developing novel therapeutic strategies. With a long half-life in the biological fluids, and their compatibility with the currently available detection, quantification and isolation methods, TEVs could be practically used for NB-diagnosis and prognosis. However, with the limitation of current detection technologies to detect low amounts of EVs released from the post-treatment residual cancer cells, compromises the application of TEVs for monitoring minimal residual disease and thus detecting the potential recurrent tumors early-on.

Besides, TEVs have shown to play prominent roles in regulating the TME and ME by interacting with immune/stromal cells, thus serving as attractive targets for designing immune-based therapies for NB. NB-EVs exhibit a differential cargo reflecting the donor cell profile, that mediate tumor-promoting effects by modulating the surrounding NB cells or stromal/immune cells. Thus, targeting the release or biosynthesis of TEVs could be a promising therapeutic approach and is an area of active investigation. However, since healthy cells release EVs for normal physiological processes, though in relatively lower amounts, the current limitation of specifically targeting the EV-release from tumor cells or pro-tumor stromal cells needs to be considered. Blocking TEV uptake and signaling in the recipient cells is another potential opportunity to inhibit NB progression and metastasis. Whereas NB-EVs have a great potential of being utilized in potential diagnostic, prognostic and clinical applications, we are only beginning to fully understand the plethora of NB-EVs function *in vivo* and especially in NB patients. In summary, with continued advances in EV research, TEVs hold great promise to be translated into clinical use with multi-purpose applications for NBs.

## Author contributions

MRD: Conceptualization, Writing – original draft, Writing – review & editing. VSS: Conceptualization, Funding acquisition, Project administration, Resources, Supervision, Writing – review & editing.

## Glossary

**Table:**

ADCC	antibody-dependent cellular cytotoxicity
AKAP12	A-kinase anchor protein 12
CAR	constitute androstane receptor
CDC	complement-dependent cytotoxicity
cfDNA	circulating free DNA
CRC	colorectal cancer
CTCs	circulating tumor cells
DCN	decorin
DLS	dynamic light scattering
ECM	extracellular matrix
EMT	epithelial to mesenchymal transition
ESCRT	endosomal sorting complex for transport
EVs	small extracellular vesicles
HIFs	hypoxia-inducible factors
HNSCC	head and neck squamous cell carcinoma
HR	high-risk
HR-NB	high-risk neuroblastoma
HUVECs	human umbilical vein endothelial cells
HVA	homovanillic acid
IGF2BP1	Insulin-like growth factor 2 mRNA-binding protein 1
ILV	intraluminal vesicles
INRG	The International Neuroblastoma Risk Group
IR	irradiation
ISEV	International society for extracellular vesicles
LDH	lactate dehydrogenase
LR-NB	low-risk neuroblastoma
LTBP1	latent-transforming growth factor-beta-binding protein 1
LUM	lumican
ME	microenvironment
MMP-9	matrix metalloproteinase-9
MRP1	multidrug resistance protein 1
MSCs	mesenchymal stromal cells
MVBs	multivesicular bodies
MVs	microvesicles
NB	neuroblastoma
NB-EVs	neuroblastoma derived small extracellular vesicles
NCAM	neural cell adhesion molecule
NCL	nucleocin
NK	natural killer
NOD1	Nucleotide-binding oligomerization domain-containing protein 1
Nrp1	Neuropilin-1
NSE	neuron-specific enolase
nSMase	neutral sphingomyelinase
NTA	Nanoparticle tracking analysis
PDAC	pancreatic ductal adenocarcinoma
PD-L1	Programmed death-ligand 1
piRNAs	P-element induced wimpy testis (PIWI)-interacting RNAs
PKM2	pyruvate kinase M2
PMN	pre-metastatic niche
PSCs	pancreatic stellate cells
ROC	receiver operating characteristic
SEMA3A	Semaphorin-3A
TAMs	tumor-associated macrophages
TEM	transmission electron microscopy
TEVs	tumor derived EVs
TME	tumor microenvironment
VASP	vasodilator stimulated phosphoprotein
VGPR	very good partial response
VMA	vanillylmandelic acid.
